# Evaluation of acoustic pulse technology as a non-antibiotic therapy for bovine intramammary infections: Assessing bacterial cure vs. recovery from inflammation

**DOI:** 10.3389/fvets.2023.1079269

**Published:** 2023-03-13

**Authors:** Shlomo Eduardo Blum, Oleg Krifuks, Limor Weisblith, Marcelo Fleker, Yaniv Lavon, Alon Zuckerman, Yochai Hefer, Omri Goldhor, Dani Gilad, Tal Schcolnic, Gabriel Leitner

**Affiliations:** ^1^Department of Bacteriology, Kimron Veterinary Institute, Bet Dagan, Israel; ^2^Israel Cattle Breeders Association, Caesarea, Israel; ^3^Gal-Yam Dairy Farm, Kfar Glikson, Israel; ^4^Nofim Dairy Farm, Gazit, Israel; ^5^Haemek Dairy Farm, Yifat, Israel; ^6^Armenta Ltd., Ra'anana, Israel

**Keywords:** acoustic pulse technology, mastitis, bacteriology, cure, recovery, dairy cow, intra-mammary infection, antibiotic alternatives

## Abstract

**Introduction:**

The spread of antimicrobial resistance (AMR) is a major threat to human and animal health. Therefore, new solutions are needed to prevent returning to a world without effective antibiotics. Mastitis in dairy cows is a major reason for antimicrobial use in food animal production, and mastitis-causing bacteria have the potential to develop AMR. In this study, acoustic pulse technology (APT) was explored as an alternative to antimicrobials for the treatment of mastitis in dairy cows. APT involves the local transmission of mechanical energy through soundwaves which stimulate anti-inflammatory and angiogenic responses in the udder. These responses promote udder recovery and enhance resistance to bacterial infections.

**Methods:**

We examined 129 Israeli dairy cows with mastitis in this prospective, controlled study to assess the efficiency of APT treatment on *cure* and *recovery* rates. An accurate diagnosis of suspected or confirmed infectious mastitis was made from cows having clinical signs of mastitis and/or somatic cell count (SCC) of above 400,000 cells/mL. The cows were divided into three groups: Group 1 (*n* = 29), cows with no bacterial findings (NBF); Group 2 (*n* = 82), cows with clinical signs of mastitis or SCC >400,000 cells/mL in the most recent test; and Group 3 (*n* = 18), cows with chronic mastitis (two or more tests with SCC >400,000 cells/mL within 3 months). All the cows received APT treatment, which involved 400 pulses on two sides of the infected quarter, delivered in three phases over 3 days. The *cure* for the mammary gland was indicated by the absence of bacterial growth in post-treatment cultures and *recovery* by a decrease in SCC to < 250,000 cells/mL in two of three post-treatment tests.

**Results and discussion:**

In Group 2, cure and recovery rates were 67.1 and 64.6%, respectively, and were not significantly different between Gram-negative and Gram-positive infections. A similar recovery rate was found in NBF cows. However, in cows with chronic mastitis, both the cure and recovery rates were significantly lower (22.2 and 27.8%, respectively). These results have important implications for dairy farmers, as APT treatment could lead to substantial savings of up to $15,106/year in a 100-cow herd, considering the national estimated prevalence of mastitis and the cost of individual treatment. APT should be further investigated as a viable and sustainable alternative to antimicrobial therapy for mastitis, offering economic benefits to dairy producers and the possibility of preventing AMR.

## 1. Introduction

Mastitis is a common disease among dairy cows and a major problem in dairy production worldwide. It is the leading cause of antibiotic use in cows, and the high prevalence of mastitis may contribute to the development of antibiotic resistance (1, 2). The main cause of mastitis in dairy cows is an intramammary bacterial infection. While various bacterial species may cause mastitis in dairy cows, most cases involve a single species. Clearing the infecting bacteria from the mammary gland, either naturally or through treatment, is key to the healing process. The term “cure” is often used to address bacterial clearance in mastitis, i.e., no bacteria were isolated in the follow-up tests after the infecting pathogen had been identified in the first test. It is used mainly to assess the success of antibiotic treatments. However, tissue damage caused by either the infecting bacteria or the inflammation process, as well as the detrimental effects of mastitis on udder function and milk quality, may persist long after bacterial clearance. Therefore, *cure* and actual *recovery* of the mammary gland to its physiological status prior to mastitis are distinct outcomes. In many cases, the mammary gland remains inflamed, with reduced milk yield and altered milk quality regardless of bacterial cure (3). Continuous decreases in milk yield and quality can often result in the farmer choosing to dry off the affected gland or even cull the animal for economic losses.

Acoustic pulse technology (APT) has been recently adapted specifically for the treatment of mastitis in dairy cows (4). APT uses repeated projectile collision with an anvil connected to the treatment head to produce low-incidence shockwaves (acoustic pulses), which are transferred non-invasively to the affected mammary gland tissues. The main goal of APT treatment is to trigger the body's self-healing mechanisms to repair damaged tissues (5). In terms of mastitis, APT mainly aims to promote the *recovery* of the mammary gland, that is, a decrease in somatic cell count (SCC) back to the normal level (< 250,000/mL) within days or weeks, without a major decrease (< 10%) in milk yield (for at least two of three follow-up monthly milk measurements) following treatment. APT also promotes increased activation of the immune system (6). Hence, a secondary goal of APT treatment is to facilitate bacterial clearance or cure. (7). Currently, antibiotics are becoming less available to farmers due to consumers' growing awareness of animal welfare and the spread of bacteria with antimicrobial resistance (AMR) (8, 9). Therefore, APT could be an alternative to antibiotic treatment in bovine to treat mastitis, especially during lactation, when antibiotics must be evidence-based to encourage their judicious use (10) or when their cost-effectiveness is limited due to the retrieval time.

The present study was built upon a previous large-scale retrospective analysis that examined data from the Israeli Dairy Herd Book, which included 123,985 cows from 650 herds. The previous study evaluated two types of mastitis recovery (i) “spontaneous recovery” from mastitis (inflammation) and (ii) recovery from mastitis following antibiotic use (11). With the SCC threshold set at < 100,000 cells/mL, only 10% of the cases showed spontaneous recovery, while with a threshold of < 250,000 cells/mL, spontaneous recovery was observed in approximately 25% of cases; no significant difference was found between the rates of “spontaneous recovery” and recovery following antibiotic treatment. In addition, a retrospective analysis was performed that compared the effectiveness of APT treatment against no treatment in the cows with subclinical mastitis in commercial dairy farms. The analysis evaluated various factors, such as cure and recovery rates, culling rates, milk yield, and economic impact. It was found that the cows treated with APT had significantly higher cure and recovery rates [Lavon et al., (11) submitted]. However, the latter study had a major limitation regarding the assessment of cure rates due to a partial lack of follow-up tests. Specifically, the cows with a decrease in SCC and no sign of mastitis were not tested by bacteriological culture for the cure. This limitation was addressed in the present study, which aimed to assess, in a prospective trial, the effect of APT treatment in cows with clinical and subclinical mastitis on the cure and recovery rates and the correlation between them.

## 2. Materials and methods

### 2.1. Study layout

The study involved 129 Israeli Holstein cows from three commercial dairy herds. These cows had clinical and subclinical mastitis, with an average of 111 days in milk and 2.8 in lactation numbers, and no differences were observed between the three herds. Herd 1 had 700 lactating cows (Hafer dairy farm, Kibbutz Haogen, Israel), herd 2 had 1,200 lactating cows (Haemek dairy farm, Kibbutz Yifat, Israel), and herd 3 had 700 lactating cows (Nofim dairy farm, Kibbutz Gazit, Israel) (farm names mentioned with consent). The cows were housed in open sheds, fed a total mixed ration of 16.8% crude protein and 1.75 Mcal/kg dry matter, and milked three times daily, yielding approximately 12,500 L of milk at 305 days of lactation. The cows were selected for the study when they showed clinical signs of mastitis or subclinical mastitis identified by increased SCC (>400,000 cells/mL) in the routine monthly milk recording of the Dairy Herd Improvement Program (DHI). The California Mastitis Test was used to identify the specifically affected quarters with subclinical mastitis. Milk samples were aseptically collected by trained farm personnel and tested at the “mastitis control” laboratory of the Israeli Dairy Board (Caesarea, Israel) and the bacteriology laboratory at the Kimron Veterinary Institute (Bet Dagan, Israel) for the presence and identification of the infecting bacteria. The cows were treated with APT and re-sampled for bacteriological culture after 20–25 days. The APT protocol involved adding 400 pulses on two sides of the infected quarter, delivered in three phases for 3 days at 2–3 day intervals (4, 12).

The analyzed data included information about the lactation number, days in milk (DIM), daily milk yield, SCC at the time of treatment, SCC and daily milk yield of the two previous and up to three monthly DHI milk tests after treatment (Herd Book of the Israeli Cattle Breeders Association, Caesarea, Israel), and management decisions regarding dry-off of infected glands or culling of cows. The cows were divided into three groups based on bacteriology results and mastitis type: Group 1 (*n* = 29), cows with clinical or recent subclinical mastitis (i.e., increased SCC in the last DHI test prior to treatment) and no bacterial finding (NBF) before treatment; Group 2 (*n* = 89), cows with clinical or recent subclinical mastitis and a bacterial finding; Group 3 (*n* = 18), cows with chronic mastitis (i.e., increased SCC in more than one DHI monthly milk test before the study). The groups were first analyzed separately to assess the *recovery* rate for each. Then, a second analysis was performed on Groups 2 and 3 to assess bacterial *cure* rates and their correlation with *recovery*. Bacterial species were grouped as either Gram-positive or Gram-negative. The *recovery* rate was calculated based on SCC at up to 3 months post-treatment, using SCC < 250,000 cells/mL as the threshold. A cow was considered “recovered” if its SCC was lower than this threshold on two out of the three test days post-treatment. The *cure* rate was defined as the absence of the bacterial species found in the milk sample before APT treatment in the same quarter, but for a more stringent analysis, even the growth of a different bacterial species was not considered a cure.

### 2.2. Statistical analysis

All statistical analyses were performed using the GLIMMIX procedure of SAS (SAS Institute, version 9.1, 2009). The analyses were conducted separately to calculate cure and recovery rates. To calculate recovery rates for all three groups (*n* = 129), we used the general form: recovery = herd + group + parity + DIM + error, where herd = the three different dairy farms, Group = 1 to 3 as described above, parity = 1st, 2nd, or 3rd and more lactations, and DIM = days in milk as a continuous effect. To calculate cure rates in Groups 2 and 3 (n = 100), we used the general form: cured = herd + Group + parity + DIM + error, where: herd = the three different dairy farms, Group = 2, 3 only, parity = 1st, 2nd, or 3rd and more lactations, and DIM = days in milk as a continuous effect. The third analysis tested cure rates according to bacterial type (Gram-negative, *n* = 23, or Gram-positive, *n* = 77) with the model: cured = bacterial type + parity + DIM + error, where bacterial type = Gram-positive or Gram-negative, parity = 1st, 2nd or 3rd, and more lactations, and DIM = days in milk as a continuous effect. Linear correlations between cured and recovered according to the bacterial type were conducted with the PROC CORR procedure of SAS (SAS Institute, version 9.1, 2009), and the data were presented as means and SEM.

## 3. Results

Neither the herd nor parity or DIM impacted cure or recovery rates. Before APT treatment, 100 cows were identified with an intramammary bacterial infection. Of these, 82 cows had clinical or recent subclinical mastitis (Group 2), 18 cows had chronic subclinical mastitis (Group 3), and 29 cows with clinical or recent subclinical mastitis had no bacterial findings (Group 1).

The bacterial cure rate at 20–25 days after APT treatment and mastitis recovery rates, including Groups 2 and 3 (*n* = 100 cows), are summarized according to bacterial species in Table 1. The overall cure rate was 59%, with a higher rate for Gram-negative bacteria (77.7%) compared to Gram-positive bacteria (40.0%) (*P* < 0.02). The overall recovery rate was similar to the cure rate, showing the same trend, with a higher rate for Gram-negative bacteria (81.0%) compared to Gram-positive bacteria (35.2%). The relationship between bacterial cure and mastitis recovery was weak, with cure and recovery occurring simultaneously in only 44% of the cows.

**Table 1:** Bacterial species found in Groups 2 and 3 (*n* = 100) before APT treatment, rates of same or different findings following treatment, respective cures at 20–25 days, and recovery rates up to 3 months.

**Bacteriology finding**	**Pretreatment (*n*)**	**Post-treatment**		**Cure^a^**	**Recovery**
		Same species isolated^c^	Different species isolated^c^		
*Escherichia coli*	22	4.5% (1)	13.6% (3)	81%	
*Pseudomonas aeruginosa*	1	100% (1)		0%	
Total Gram-negative	**23**			**78.3% (18)**	**82.6% (19)**
*Streptococcus dysgalactiae*	15	26.7% (4)		73.3%	
*Streptococcus uberis*	15	46.7% (7)	13.3% (2)	40%	
*Enterococcus* spp.	7	85.7% (6)		14.3%	
Total *Streptococcus* spp.	**37**			**48.6% (18)**	**43.2% (16)**
*Staphylococcus haemolyticus*	14	57.1% (8)		42.8%	
*Staphylococcus chromogens*	10	30% (3)	50% (5)	20%	
Other coag-neg. *Staphylococcus*^b^	14	14.3% (2)		85.7%	
Total *Staphylococcus* spp.	**38**			**52.6% (20)**	**57.9% (22)**
*Trueperella pyogenes*	2		100% (2)	0%	
Total Gram-positive	**77**			**49.4% (38)**	**49.3% (38)**
Total	**100**			**56% (56)**	**57% (57)**

^a^Cure = No bacteria were isolated following treatment.

^b^*Staphlococcus epidermidis* (3), *Staphlococcus hyicus* (1), *Staphlococcus simulans* (1), *Staphlococcus microti* (1), and unidentified coagulase-negative staphylococci (8).

^c^Percentage and number of cases in which the same bacterial species listed in “Bacteriolgy finding” was isolated after treatment or a different species.

Bold values are a total for the corresponding pathogen group.

The second analysis examined each group separately because there was an interaction between Groups 2 and 3 and Gram-negative and Gram-positive findings. The overall cure and recovery rates for Group 2 were 67.1 and 64.6%, respectively, exhibiting a weak relationship (0.35, Table 2). The cure rates for Gram-negative and Gram-positive bacteria (85.7 and 60.7%, respectively) were not statistically significant (*P* > 0.05), and the same was true for the recovery rates (76.2 and 60.7%, respectively). The results for recovery in Group 1 (NBF) were similar, with a rate of 72.4%. The cure rate could not be determined due to the absence of bacterial isolation prior to treatment. In contrast, the cure and recovery rates for Group 3 (chronic subclinical mastitis) were significantly lower, at 22.2 and 27.8%, respectively, with a correlation of 0.56. Only Gram-positive bacteria were isolated from the cows in Group 3.

**Table 2:** The results of the generalized mixed model used to estimate the effect of APT treatment on cure and recovery rates in the cows (*n* = 129, 3 herds) with clinical or subclinical mastitis caused by Gram-negative or Gram-positive bacteria or without bacterial findings.

			**Cure** ^**a**^				**Recovery** ^**b**^			
**Variable**	**Level**	**Cows #**	**LSM diff** ^*c*^	**REF level** ^*d*^	***P*** **-value**	**SE**	**LSM diff**	**REF level**	***P*** **-value**	**SE**
Herd	1	18	-	0.667	-	-	-	0.709	-	-
	2	41	−0.188		0.967	0.136	−0.107		0.685	0.1
	3	41	−0.009		1	0.13	−0.174		1	0.095
Parity	1	25	-	0.554	-	-	-	0.694	-	-
	2	19	0.104		1	0.16	−0.08		1	0.117
	3+	56	−0.043		1	0.114	−0.152		0.559	0.082
Bact_BT^*e*^	NBF	-	-	-	-	-	-	0.724	-	-
	Gram+	78	-	0.4	-	-	−0.186		0.54	0.057
	Gram-	22	0.377		0.019	0.039	0.003		0.99	0.097
Group	1	-	-	-	-	-	-	0.724	-	-
	2	82	-	0.81	-	-	−0.078		0.954	0.053
	3	18	−0.458		0.005	0.181	−0.446		0.05	0.109

^a^Cure: No bacteria were isolated following treatment.

^b^Recovery: Decrease in SCC in 2 out of 3 monthly tests post-treatment to < 250,000 cells/mL.

^c^Values in the Least Square Means (LSM) column are deviations from the reference levels.

^d^Reference levels for comparison of cure or recovery rates.

^e^Bact_BT: bacteriological finding before treatment.

Subclinical mastitis was determined by the pattern and level of SCC with a threshold of 400,000 cells/mL.

**Figure 1:**
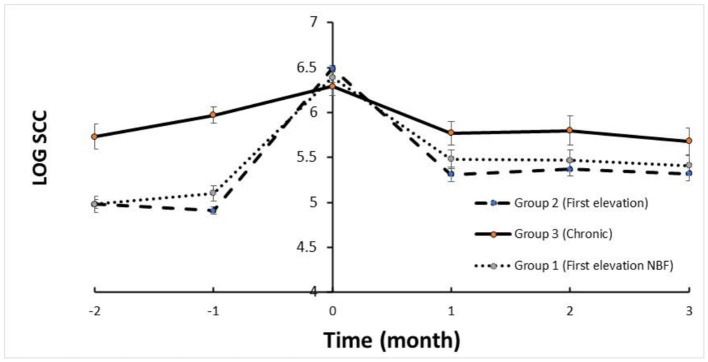
Mean log of SCC relative to APT treatment according to groups.

**Figure 2:**
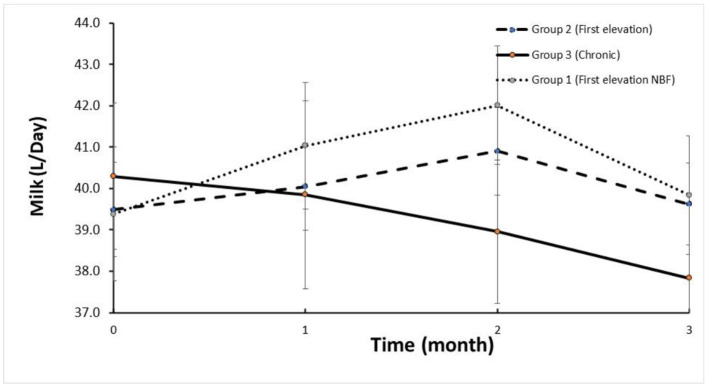
Average daily milk yield (L/day) relative to APT treatment according to groups.

The trends in SCC are depicted in Figure 1. The mean SCC before treatment in Group 2 and Group 1 was < log 5, increasing to >log 6 and returning to log < 5.5 (i.e., < 250,000 cells/mL) for the three DHI monthly milk recordings. Milk yield in these groups increased after treatment within 2 months following treatment (Figure 2). In contrast, the trend in SCC and milk yield was different for Group 3 (chronic subclinical mastitis). The mean SCC in the 2 months before treatment was >log 6; on average, SCC levels were similar after and before treatment. However, daily milk yield gradually decreased after treatment.

## 4. Discussion

The main factors affecting the successful treatment of mastitis are pathogen species, host factors such as genetics, the immune and physiological status that affect the host's ability to cope with the infecting pathogen, and the time from infection establishment to treatment initiation. “Spontaneous cure”—when the pathogen is eliminated by the mammary gland immune system without external intervention—occurs only in ~20% of cases (11), but this rate varies greatly depending on the infecting bacteria. Antibiotic therapy is the most common method of eliminating intramammary infections (1, 2, 13) with various degrees of success. However, the extensive use of antibiotics in treating and preventing intramammary infections may contribute to the development of AMR (14). With increasing consumer awareness and regulatory action aimed at preventing the spread of AMR bacteria, such as the European Union's strategies and action plans for reducing antibiotic overuse (15), antibiotics are becoming less available for use in livestock production, including mastitis therapy (9, 16).

Moreover, due to the high cost of antibiotic treatment during lactation (due to the need to discard milk for the retention period), many cows without a life-threatening infection are treated only at dry-off, thus becoming chronically infected. In these chronically infected cows, tissue damage worsens over time, resulting in lower milk yield and quality (17–20). These cows are the hardest to treat, and even antibiotic therapy often fails to clear the infection.

In the present study, we emphasized the difference between bacterial ***cure*** and ***recovery*** from mastitis. Cure relates to the clearance of infection, often measured as a lack of bacterial isolation in follow-up tests, for example, after treatment. Cure rates reported following antibiotic therapy differ greatly (from 4 to 92%) depending on the pathogen species, the antibiotics studied, and the methods used to detect and follow-up intramammary infections (and their cure), such as the number of samplings and the laboratory technique used for the isolation and identification of pathogens (21). When the cause of mastitis was Gram-negative bacteria, antimicrobial treatment did not improve the outcome of the infection (22, 23).

Healing (recovery) is usually less studied. In the context of mastitis, recovery can be measured by inflammation parameters, with SCC being the most common and well-established one. Because SCC is also used as a milk quality parameter, it has a direct economic impact on the farm. The mammary gland often remains inflamed for a long time following mastitis, regardless of the presence of the infecting bacteria that triggered the inflammation in the first place (24, 25). This, in turn, leads to prolonged lower milk yield and quality and the eventual need to dry off the affected quarter or even cull the cow, based on the farmer's cost-benefit judgment (26–28). In a review by Ruegg (29), it was concluded that “associations between bacteriological cure and clinical outcomes are very weak” and “evaluation of continued decline in quarter-level SCC appears to be the most reliable indicator of success.” If the mammary gland tissue has suffered extensive destruction or damage caused directly by the pathogen or the inflammation, milk production and its quality can be affected for a long time despite a bacterial cure. Thus, even though bacterial clearance from the mammary gland is key to healing, it does not ensure the total recovery of the gland's physiological status.

The effectiveness of using APT to treat mastitis in dairy cows has been recently explored (4, 7, 12, 11, submitted), with the major goal of promoting recovery from inflammation rather than curing an intramammary infection. The results in these studies showed 65–75% recovery of cows, which is similar to the results of the present study for Groups 1 and 2 (clinical and recent subclinical mastitis, either with or without detected infection). Although cure was attained in most cows in Group 2 (67%), we found a low correlation with actual recovery (0.35). In ~14% of the cows in this group, bacterial infection was still detected, regardless of the recovery of the gland (based on the parameters of udder health used here), whereas in approximately 14% of the cows, no infection was further detected, but mammary gland health did not return to normal. In this study, the importance of the timing of treatment is emphasized by the results observed in Group 3, which included cows with chronic subclinical mastitis for at least 3 months. In these cows, both the cure and recovery rates were significantly lower (about 27%). Moreover, the significant difference in cure rates in cases caused by Gram-positive bacteria between Groups 2 and 3 (i.e., between clinical or recent subclinical and chronic mastitis) highlights the notion that timing of mastitis identification and prompt treatment may be more important factors than bacterial type.

The results of the present study indicate that APT not only promotes tissue recovery after inflammation but also aids in bacterial clearance, helping to decrease the extent of tissue damage in the mammary gland and limit the spread of pathogens between cows and into the milk tank. A bacterial cure may be due to activation of the immune response, increased vascularization in the mammary gland, or both. Thus, APT could be of value to the farms' current mastitis control programs and improve milk quality. For instance, an economic cost-benefit analysis was performed for treating subclinical mastitis with APT compared to no treatment [Lavon et al., (11) submitted]. For this estimate, the milk yield of a regular Israeli herd was estimated at 40 L/day on average, or 12,200 L/305 days, with an 18% prevalence of subclinical mastitis. The milk price was fixed at USD$ 0.32/L, and the price for a replacement heifer was fixed at USD$ 1,500. Under these parameters, the present findings suggest that APT could save up to USD$ 15,106/year in mastitis control costs in a 100-cow herd.

The results of the present study highlight the importance of timely treatment of mastitis in dairy cows, as delays between detection and treatment, can significantly impact the success rate of the treatment. As APT is non-invasive and does not involve antibiotics, treatment can be performed during lactation soon after the detection of mastitis, thus potentially increasing the likelihood of success. Timely treatment can reduce the risk of antibiotic residues in milk and the need to dispose of waste milk. Additionally, early treatment can help reduce economic losses due to elevated SCC levels in bulk milk and improve animal welfare throughout the lactation period. Furthermore, early mastitis treatment can decrease the need for antibiotics later on during dry-off therapy, reducing the overall use of antibiotics in dairy production.

In addition to milk quality and safety for human consumption, the use of antibiotics to treat mastitis can affect the development and shedding of antibiotic-resistant bacteria in a herd. Previous research suggests that dry-cow therapy may increase the levels of antibiotic-resistant fecal bacteria in dairy cows (30). Furthermore, it is a common practice to feed calves waste milk, including milk that had to be discarded due to antibiotic treatment during lactation. Existing evidence shows that this can lead to an increase in the shedding of ESBL-producing *Escherichia coli* in calves (31). Hence, the risk of increasing the burden of antimicrobial resistance (AMR) due to antibiotic use for mastitis is multifaceted. Non-antibiotic alternatives to treat mastitis in dairy cows that reduce antibiotics in dairy products are beneficial in combating AMR from a broader “One Health” perspective. Selective dry-off therapy, which involves administering antibiotics only to infected mammary glands at the end of lactation, is one tool that has been proven to decrease the overall use of antibiotics in dairy farms (32). APT may have similar benefits. However, as APT is not an antibiotic, it does not necessarily require bacterial culture results for application. Therefore, APT should be explored further as an alternative therapy for mastitis in dairy cows.

## Data availability statement

The raw data supporting the conclusions of this article will be made available by the authors, without undue reservation.

## Ethics statement

The animal study was reviewed and approved by Kimron Veterinary Institute institutional committee for ethical animal experimentation. Written informed consent was obtained from the owners for the participation of their animals in this study.

## Author contributions

Study design, data analysis, and manuscript writing: SB and GL. Field work: AZ, YH, OG, DG, and TS. Laboratory work: OK, LW, and MF. Statistical analysis and graphics design: YL. All authors contributed to the article and approved the submitted version.
